# Are Systematic Screening for Vitamin D Deficiency and Vitamin D Supplementation Currently Feasible for Ankylosing Spondylitis Patients?

**DOI:** 10.1155/2017/7840150

**Published:** 2017-01-01

**Authors:** Mickael Essouma, Jean Jacques N. Noubiap

**Affiliations:** ^1^Department of Internal Medicine and Specialties, Faculty of Medicine and Biomedical Sciences, University of Yaoundé I, Yaoundé, Cameroon; ^2^Department of Medicine, Groote Schuur Hospital and University of Cape Town, Cape Town, South Africa

## Abstract

Beyond its role in calcium and phosphorus metabolism for healthy bone mineralization, there is increasing awareness for vitamin D contribution in modulation of immune reactions. Given that ankylosing spondylitis (AS) is a chronic inflammatory disease involving excess immune/inflammatory activity and posing great therapeutic challenges, it is conceivable to claim that vitamin D treatment may be a safe and effective treatment to influence or modify the primary disease and its related comorbidities. Nevertheless, consistent body of research supporting this hypothesis is still lacking. In this paper, we examine whether systematic screening and treatment for vitamin D deficiency are feasible at present. We will review the immunomodulatory role of vitamin D and its contribution in initiation and progression of AS, as well as how they would determine the occurrence of comorbid conditions. Our conclusion is that despite the overwhelmed interest about vitamin D treatment in AS patients, systematic screening and treatment for vitamin D deficiency of all AS patients are not feasible as yet. This stresses the need for further extensive well-designed research to prove vitamin D efficacy in AS beyond bone protection. And if utility is proven, personalized treatment regimes, duration of treatment, and threshold values for vitamin D should be provided.

## 1. Introduction

Ankylosing spondylitis (AS) is a chronic inflammatory disease of the axial spondyloarthritis (SpA) family with prominent involvement of the spine and sacroiliac joints resulting in formation of syndesmophytes that progressively lead to bony ankylosis of the spine [1]. Other musculoskeletal features suggestive of AS include peripheral arthritis and enthesitis, whereas extra-articular features are uveitis, cardiac diseases including valve insufficiency and heart blocks, lung disease such as upper pulmonary lobe fibrosis, gastrointestinal disease which is mostly subclinical inflammatory bowel disease, retroperitoneal fibrosis, and skin disease [2]. The global prevalence of AS varies between 0.02% and 0.35% [3]. The risk and progression of AS are determined by both genetic and environmental factors. Among genetic factors, the human leukocyte antigen- (HLA-) B27 is the strongest one even though the overwhelming majority of these genes are outside the major histocompatibility (MHC) molecules along with other HLA-B alleles [4]. Environmental factors mainly include tobacco smoking [5]. The initial presentation usually occurs between the ages of 30–45 years, with a sex ratio of 3.4 : 1 (males : females) [6]. AS progression results in reduced functional capacity which is further worsened by comorbidities especially cardiovascular diseases and osteoporosis [7–9]. To limit AS progression, a large array of pharmacological treatments are currently used, nonsteroidal anti-inflammatory drugs being the mainstay and tumor necrosis factor inhibitors (TNFi) the best suitable alternative in nonresponsive patients unless there is an absolute contraindication [10, 11].

Vitamin D is a secosteroid hormone that contributes to calcium and phosphorus metabolism for healthy bone mineralization [12]. Vitamin D includes vitamin D_2_ (derived from plants) and vitamin D_3_ (derived from animals) as well as vitamin D metabolites. Most of the data from this paper focus on vitamin D_3_ (also known as cholecalciferol) given that vitamin D_3_ appears to be more effective than vitamin D_2_ (also known as ergocalciferol) [13]. In humans, ~90% of vitamin D arises from endogenous synthesis by conversion of 7-dehydroxycholesterol into previtamin D by ultraviolet B radiations and then to vitamin D by a nonenzymatic transformation. Considering that vitamin D is biologically inactive, it is activated in a two-stage hydroxylation process: first, hepatic hydroxylation that results in 25-hydroxyvitamin D (25[OH]D) and, second, hydroxylation of 25(OH)D in the kidneys, resulting in 1,25-(OH)_2_D which is the active form of vitamin D [12, 14].

Recent insights have shed light on supplemental roles of vitamin D. For instance, functional laboratory studies have shown the involvement of vitamin D in modulation of immune and inflammatory reactions. Along the same lines, epidemiological studies involving patients with numerous chronic inflammatory diseases including rheumatism have repeatedly suggested its implication in the occurrence and worsening of those diseases [12–15].

With respect to the relatively short half-life of 1,25(OH)_2_D (~4 hours), vitamin D status is best monitored by 25(OH)D which is the major circulating form of vitamin D with a long half-life (2-3 weeks). 25(OH)D status has been defined by various groups in the literature. Whatever the definition chosen is, levels of 25(OH)D defining vitamin D deficiency/insufficiency are retained on the basis of either a clinical or a biological parameter [16]. Notably, the fracture risk in elderly subjects is the main suggestive clinical parameter whereas biological criteria are elevated serum parathyroid hormone and increased bone turnover markers. In clinical practice, definitions commonly used are those provided by both the Institute of Medicine (IOM) and the Endocrine Society. According to both groups, vitamin D deficiency is defined as 25(OH)D below 20 ng/ml, insufficiency as 25(OH)D of 21–29 ng/ml, and sufficiency as 25(OH)D of 30–100 ng/ml [16, 17].

Although there is much excitement about vitamin D in the context of systemic inflammation and immune reactions and AS has been associated with low 25(OH)D in observational studies [18–20], the benefits of a putative treatment with vitamin D in AS patients beyond bone health are still theoretical. Notably, whether or not all AS patients should be screened and treated for vitamin D deficiency and specific treatment regimens remain unknown. Moreover, can that treatment positively influence the course of AS and related comorbid conditions beyond bone health? This review will examine in the existing literature if systematic screening and treatment for vitamin D deficiency in all AS patients are realistic.

## 2. Epidemiology of Vitamin D Deficiency in “AS”

Serum levels of 25(OH)D have been determined in AS patients and compared with healthy subjects in a number of cross-sectional studies (Table 1) [18, 19, 21–28]. The Swedish study by Klingberg et al. found no difference in serum 25(OH)D of 203 AS patients compared with 120 healthy subjects in the late winter season [21]. Nevertheless, there were significantly more users of vitamin D supplements in the AS group compared with healthy controls, what may have masked any difference. By contrast, a recent systematic review summarizing evidence from eight cross-sectional studies totalizing 555 AS patients compared with 557 healthy controls found over all studies serum mean 25(OH)D of 22.8 ± 14.1 ng/ml in AS patients and 26.6 ± 12.5 ng/ml in healthy controls. Furthermore, 25(OH)D levels were significantly higher in healthy controls (3.8 ± 0.8 ng/ml, *p* < 0.01) [29].

## 3. Mechanistic Link between Low Vitamin D Status and “AS”

### 3.1. Immunomodulation by 1,25-Dihydroxyvitamin D

Experimental studies have shown that 1,25(OH)_2_D acts through the nuclear vitamin D receptor (VDR) which is found in most tissue cells including inflammatory cells, especially in macrophages, dendritic cells, and B as well as T lymphocytes [30].

1,25-Dihydroxyvitamin D can promote monocyte-to-macrophage differentiation and induce the production of immunosuppressant cytokines (e.g., prostaglandin E2) [31]. Conversely, 1,25(OH)_2_D is a potent downregulator of proinflammatory cytokines and chemokines, namely, tumor necrosis factor alpha (TNF-*α*), interleukin-1 (IL-1), interleukin-6 (IL-6), interleukin-17 (IL-17), and interleukin-23 (IL-23) [31, 32]. Furthermore, 1,25(OH)_2_D can impair macrophage-related antigen presentation by reducing the expression of class II MHC molecules upon their surface [32].

1,25-Dihydroxyvitamin D is also capable of inhibiting monocytes' maturation and differentiation into dendritic cells (DC). This results from downregulation of nuclear factor kappa B and subsequent T helper (Th) 1 response suppression [14]. 1,25(OH)_2_D may affect the DC/T cell interaction via action on costimulation molecules, thus decreasing the production of proinflammatory cytokines (e.g., IL-1 and TNF-*α*) and membrane expression of class II MHC molecules [30]. In addition, 1,25(OH)_2_D upregulates CD4+CD25+T regulatory cells (Treg) probably by increasing the production of Fox-P_3_ and interleukin-10 (IL-10) [33, 34].

**Figure 1:**
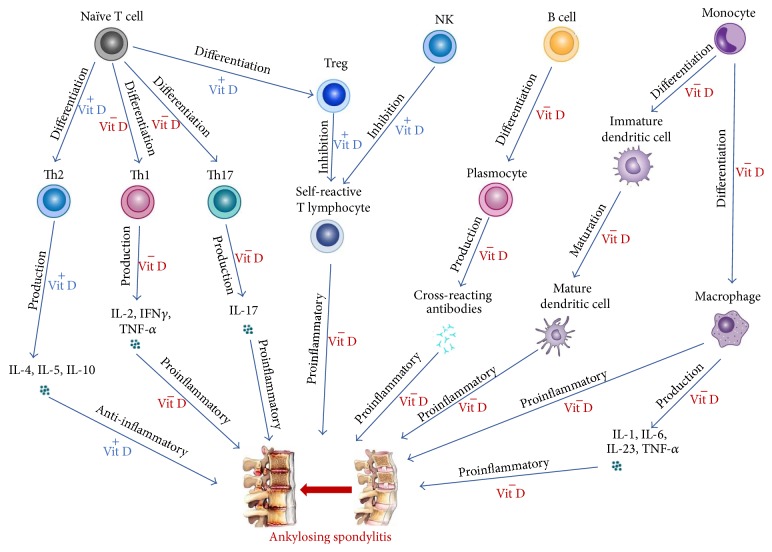
Vitamin D immunomodulatory activity influencing ankylosing spondylitis. +: stimulation by vitamin D (Vit D); −: inhibition by Vit D. Vit D can have effect on the naïve T cell, the natural killer (NK) cell, the B cell, and monocyte hence putatively inhibiting ankylosing spondylitis pathogenesis. (I) Vit D may stimulate the naïve T cell's differentiation into T helper (Th) 2 with raised production of anti-inflammatory cytokines and into the T regulatory (Treg) cell, thus inhibiting the self-reactive T lymphocyte. Besides, Vit D may inhibit differentiation of the naïve T cell into Th1 and Th17 with decreased production of proinflammatory cytokines. (II) Vit D may stimulate the NK cell to inhibit the self-reactive T lymphocyte. (III) Vit D may inhibit differentiation of the B cell into plasmocyte, thus inhibiting the production of cross-reacting antibodies. (IV) Vit D may inhibit monocyte-to-dendritic cell differentiation and monocyte-to-macrophage differentiation, with consequential reduced production of proinflammatory cytokines. IL: interleukin; IFN*γ*: interferon gamma; TNF-*α*: tumor necrosis factor alpha.

Besides, 1,25(OH)_2_D alters both B and T cell immune actions. For instance, in the case of B cells, acknowledged effects of 1,25(OH)_2_D are mostly limited to downregulation of their proliferation, differentiation to plasma cells, and immunoglobulin production. Concerning effector T cells, their immune response is suppressed both directly by inhibition of T cell proliferation and indirectly via DC inhibition and blockade of DC/T cell interaction. Suppression of T cell proliferation results in downregulation of Th1 and Th17 responses and stimulation of Th2 responses raising blood levels of anti-inflammatory cytokines (e.g., IL-10). Besides Th2 upregulation, 1,25(OH)_2_D promotes Treg and Tr1 immunomodulating responses [30]. Altogether, 1,25(OH)_2_D may modulate immune reactions by altering immune cells production of proinflammatory/anti-inflammatory cytokines and by impairing their interactions (Figure 1).

### 3.2. Low Vitamin D Status and Risk and Severity of “AS”

Vitamin D deficiency has been associated with AS [20, 29, 35]. However, there is only indirect evidence to support a putative role for 1,25(OH)_2_D in the inception of AS so far. Indeed, AS is a classic inflammatory arthritis characterized by excess production of proinflammatory cytokines, especially TNF-*α*, IL-6, IL-17, and IL-23. IL-6 is largely involved in the production of acute phase reactants including C-reactive protein (CRP) and initiation as well as maintenance of inflammation [4]. Besides, adaptive T cell immunity (especially Th17 and CD_8_ T cell responses) is crucial for initiation and progression of AS [4, 5]. Regarding 1,25(OH)_2_D as a major contributor in immune responses that may largely alter the production of the aforementioned proinflammatory cytokines as well as antigen presentation (largely involved in T cell response) and so downregulate Th1 and Th17 immunity, one can speculate that in case of vitamin D deficiency excess immune responses might not be prevented and AS features would hence occur and evolve [4, 5, 30].

The relationship between AS disease activity and vitamin D deficiency is conflicting in epidemiological studies as yet. While some studies have demonstrated a significant negative correlation between AS disease activity markers and vitamin D deficiency [18, 19, 22, 25, 27], other studies have not found any correlation [21, 22, 24, 36]. Of note, disease activity markers have extensively been assessed in all those studies, from less sensitive and specific disease activity markers including CRP and erythrocyte sedimentation rate (ESR) to more specific markers, that is, Bath AS Disease Activity Index (BASDAI) and Bath AS Functional Index (BASFI). Even though most of those studies that did not find any correlation had risk of type 2 statistic errors [20], the correlations found were weak in most of the other studies [29]. Furthermore, none of those studies completely adjusted for important confounding factors. Moreover, most studies reported results of 25(OH)D using mean standard deviation; meanwhile, 25(OH)D is not a normally distributed variable [20]. Consequently, results from currently available meta-analyses need to be further explored [20, 29, 37]. Taken collectively, causation is not currently proven despite some evidence for increased risk of AS in subjects with vitamin D deficiency.

### 3.3. Klebsiella, “AS,” and 1,25-Dihydroxyvitamin D

There is overwhelming evidence from genetic, microbiological, molecular, and immunological studies supporting the crucial role of* Klebsiella* microbes in the initiation and perpetuation of AS through the molecular mimicry or cross-reactivity pathogenic processes [38]. For instance, (i)* Klebsiella* microbes were isolated more frequently from the bowel of patients with active AS and their isolations were associated with clinical exacerbations of the disease; (ii) elevated levels of anti-*Klebsiella* antibodies were observed in the sera and jejunum fluids of AS patients when compared with matched healthy controls; (iii) molecular similarity was identified between* Klebsiella* nitrogenase reductase and HLA-B27 and between* Klebsiella* pullulanase and collagen types I, III, and IV; and (iv) in vivo cross-reactivity and cellular binding and in vitro cytotoxic activities of anti-*Klebsiella* antibodies were observed in patients with AS [38].

Anti-*Klebsiella* antibodies are therefore implicated in the inflammatory processes leading to AS through cross-reaction with self-antigens in joints. As 1,25(OH)_2_D inhibits B cell differentiation into plasma cells and downregulates immunoglobulin production, especially IgM and IgG [39], it may reduce the proinflammatory effects of anti-*Klebsiella* antibodies in AS.

## 4. Vitamin D Deficiency and “AS” Comorbidities

### 4.1. Osteoporosis

This is an established and frequently undiagnosed and untreated complication of AS prevalent in up to 62% patients. The high risk of osteoporosis and related vertebral fractures seen in AS patients is determined by multiple factors including chronic inflammation, reduced motion in relation to pain and stiffness, and drug intake [36, 40–42]. Vitamin D deficiency is a common risk factor for osteoporosis in the general population [43]. In the AS population, the prevalence of osteoporosis seems particularly high in subgroups of patients with lower 25(OH)D compared to AS patients with normal 25(OH)D levels [22, 27]. Nevertheless, the true relationship between vitamin D deficiency and osteoporosis in AS patients still eludes researchers. Arends et al. indicated low 25(OH)D to be a predictive factor for low bone mineral density (BMD) and an independent relationship was found between low 25(OH)D and bone turnover markers in a Dutch cross-sectional study involving 128 AS patients [36]. In a comparative study involving 70 male AS patients and 140 controls, Hmamouchi et al. found a significant negative correlation between serum levels of 25(OH)D and BASFI (*r* = 0.22, *p* < 0.001). BASFI was positively correlated with CRP and ESR (*r* = 0.39, *p* < 0.05 and *r* = 0.36, *p* < 0.05, resp.). In addition, BASFI was positively correlated with lumbar spine BMD and femoral total BMD (*r* = 0.31, *p* < 0.001 and *r* = 0.32, *p* < 0.001, resp.). Briefly, this study suggested that vitamin D deficiency might indirectly lead to osteoporosis via upregulation of inflammatory activity [22]. Along the same lines, Lange et al. observed that high disease activity in AS is associated with impaired vitamin D metabolism and excess bone resorption [27]. Similarly, Obermayer-Pietsch et al. suggested that Fok1 polymorphism of the VDR gene is intimately associated with inflammatory activity, bone metabolism, and BMD [26]. Altogether, vitamin D deficiency in AS patients might indirectly and continuously enhance bone resorption via perpetration of inflammatory activity. This subsequently leads to osteoporosis.

### 4.2. Cardiovascular Diseases

AS patients have a 1.6–1.9-fold increased risk for cardiovascular diseases (CVD) compared with the general population [44]. Known determinants of this high cardiovascular risk are traditional CVD risk factors (hypertension, dyslipidemia, diabetes, smoking, and obesity), chronic inflammation, and disease-modifying antirheumatic drugs (DMARDs). It is noteworthy that vitamin D deficiency is a newly acknowledged risk factor for CVD in the general population. Indeed, vitamin D deficiency might be associated with endothelial dysfunction and subsequent atherosclerosis, as well as hypertension. This has been attributed to stimulation of the renin-angiotensin-aldosterone system and enhancement of proinflammatory and prothrombotic status [45]. Regarding the fact that vitamin D deficiency may be associated with the occurrence and worsening of systemic inflammation in AS and that AS is related with a high risk for CVD, it is conceivable that vitamin D deficiency could increase the risk of CVD in AS patients.

## 5. Vitamin D Treatment for “AS” Patients

### 5.1. Indications

To date, screening for vitamin D deficiency is strongly recommended only in groups of people with confirmed high risk for vitamin D deficiency in whom response to optimization of vitamin D status is expected [13, 16]. These include patients with underlying conditions (rickets, osteomalacia, osteoporosis, chronic kidney disease, hepatic failure, malabsorption syndromes, hyperparathyroidism, granuloma-forming disorders, and some lymphomas) or taking medications that interact with vitamin D metabolism (antiseizure medications, glucocorticoids, AIDS medications, ketoconazole, and cholestyramine), obese subjects, pregnant and lactating women, and older adults with history of falls or nontraumatic fractures as well as African-American and Hispanic adults and children [16].

While it is certain that supplements with vitamin D may be beneficial in AS patients presenting with either of the aforementioned conditions, current evidence does not support systematic supplementation with vitamin D of the whole AS population. Indeed, a strategy that would recommend systematic screening of all AS patients for vitamin D deficiency has not been proven feasible or cost-effective. In addition, whether such a strategy will be beneficial in terms of important health outcomes beyond skeletal benefits is still a matter of debate. However, numerous available epidemiological studies have suggested that sufficient 25(OH)D level may be beneficial in reducing the risk of AS [20, 29, 37]. Even though this is not the guarantee for cost-effectiveness of AS prevention and treatment by vitamin D supplementation, we would suggest systematic screening of vitamin D deficiency for all AS patients and treatment with vitamin D in case of vitamin D insufficiency/deficiency or if the patient has either of the ascertained risk factors for vitamin D deficiency. Focusing on the immunomodulatory role of vitamin D, systematic supplementation with vitamin D of all AS patients (irrespective of their vitamin D status) might be considered an effective add-on DMARD (alongside NSAIDs and TNFi) to reduce disease activity and comorbidities if proven effective for this indication in the future.

### 5.2. Dosages

Both vitamin D_2_ and vitamin D_3_ can be used for the treatment and prevention of vitamin D deficiency. Daily vitamin D requirements for the prevention of vitamin D deficiency vary in normal individuals by age, sex, and physiological state (lactation, pregnancy) [13, 16]. There is no universal consensus regarding these requirements at present and clinical practice is generally based on both the Institute of Medicine (IOM) and the Endocrine Society recommendations [17, 18, 46]. Several recent studies have suggested that the recommended dietary allowances for treatment and prevention of vitamin D deficiency of the IOM may not be realistic, especially for patients who have underlying conditions or are taking drugs that affect vitamin D metabolism. Hence, the recommended treatment regimens for vitamin D deficiency summarized in Table 2 are from the Endocrine Society clinical practice guideline [16]. Vitamin D may be administered every day or once a week and therapy often begins with a loading dose for many weeks followed by a maintenance dose for many years. Notably, vitamin D treatment usually appears beneficial after up to six years of treatment without discontinuation. Of course, obese adults as well as patients receiving drugs that affect vitamin D metabolism require at least two to three times more vitamin D than doses provided here. In patients with hyperparathyroidism, vitamin D should be prescribed as needed; and in patients with granuloma-forming diseases, close monitoring of calcium during vitamin D therapy is necessary given the increased sensitivity for vitamin D and the risk of toxicity [16].

Currently, there are no specific caveats guiding either on 25(OH)D target value or on appropriate treatment regimen for vitamin D deficiency in the AS population. As a result, only AS patients with concurrent ascertained risk factors for vitamin D deficiency or those with confirmed vitamin D deficiency/insufficiency might be treated with vitamin D following the Endocrine Society guidelines in absence of thorough evidence-base supporting specific treatment modalities for AS patients. In brief, future studies should focus on vitamin D treatment regimens specific to the AS population if proven necessary.

### 5.3. Safety

Vitamin D therapy is usually safe and its toxicity is extremely rare due to the wide therapeutic index of vitamin D treatment, the tightly regulated synthesis of 1,25(OH)_2_D, and the characteristics of the adipose tissue which stores and slowly releases vitamin D [13, 16, 47, 48]. Although granulomatous disorders and 24-hydroxylase deficiency as well as Williams syndrome increase sensitivity to vitamin D and potential toxicity, excessive vitamin D intakes (especially from food supplements) raising blood levels of 25(OH)D above 150 ng/ML (as suggested by the Endocrine Society) are the main cause of vitamin D toxicity [13, 16, 48–50]. Symptoms of vitamin D toxicity mainly arise from hypercalcemia and include headaches, nervousness, arthralgia, loss of appetite, nausea, vomiting, constipation, frequent urination, excess thirst, kidney stones, and itching. Broadly, vitamin D toxicity (via hypercalcemia) is mainly responsible for systemic complications involving the skeleton, the nervous system, the digestive system, the cardiovascular system (with increased CVD risk), and kidneys (with the formation of nephrolithiasis) as well as water exchange [48, 51]. Treatment of vitamin D toxicity consists of cessation of vitamin D intake and correct rehydration as well as administration of diuretics to increase urinary excretion of calcium [13, 16, 48].

Despite the fact that vitamin D toxicity often appears anecdotal, it should not be ignored. Therefore, caution should be exercised when vitamin D is systematically administered to AS patients at large given the uncertainties regarding appropriate doses and in order to avoid toxicity.

## 6. Conclusion

According to the existing literature, it is not recommended to systematically screen and treat AS patients for vitamin D deficiency. Based on current guidelines, only AS patients with confirmed risk factors for vitamin D deficiency would benefit from such a strategy. While experimental studies conclude that vitamin D is an immunomodulatory and anti-inflammatory molecule, human studies of vitamin D in relation to AS are still inconclusive. Is vitamin D deficiency really causative for AS or does it simply reflect enhanced systemic inflammation and poor health status in AS patients? The answer to this question is still incomplete. Any suggested additional effect of vitamin D beyond bone protection thus remains theoretical and we lack strong evidence that can help make specific guidelines in either the scope of vitamin D testing or ideal treatment modalities. Nevertheless, there remains considerable interest for vitamin D supplementation in the treatment of AS. Future longitudinal prospective studies and well-designed randomized controlled trial need to clarify whether vitamin D supplementation can be used as add-on DMARD to influence or modify AS and related comorbidities beyond its role in bone health. If proven effective, threshold values and personalized therapeutic regimes as well as treatment duration should be assessed and adopted.

## Figures and Tables

**Table 1:** Published cross-sectional studies assessing vitamin D deficiency prevalence in AS patients versus healthy subjects.

Author, year of publication, and location	Definition^§^	Sample size		Groups matching for			Vitamin D supplementation	Results
		AS	Controls	Age	Gender	BMI		
Klingberg et al., 2016, Sweden [21]	Insufficiency < 50 nmol/l Deficiency < 25 nmol/l	203	120	No	No	No	Not excluded	No significant difference between AS patients and HCs (51 nmol/l [IQR 37.0–67.0] versus 45.0 nmol/l [IQR 32.0–59.0], *p* = 0.044 for serum 25[OH]D and 11 ± 5.4% versus 12 ± 10%, *p* = 0.122 for deficiency)

Hmamouchi et al., 2013, Morocco [22]	Insufficiency 20–30 ng/ml Deficiency < 20 ng/ml	70	140	Yes	No	Yes	Not excluded	Significant lower levels of 25(OH)D in AS patients compared with HCs (17.5 ± 9.7 ng/ml versus 21.9 ± 7.7 ng/ml, *p* < 0.001) and significant deficiency in AS patients compared with HCs (62 [88.6%] versus 57 [40.7%], *p* < 0.001)

Erten et al., 2013, Turkey [18]	Insufficiency < 30 ng/ml Deficiency < 10 ng/ml	48	92	NR	NR	NR	Not excluded	Significant reduced vitamin D levels in AS patients compared with HCs (*p* = 0.004) Insufficiency significantly more prevalent in AS patients than in HCs (38 [80%] versus 57 [62%], *p* < 0.001) Deficiency significantly more prevalent in AS patients than in HCs (8 [17%] versus 4 [4%], *p* < 0.001)

Yazmalar et al., 2013, Turkey [23]	NR	72	70	No	No	No	Not excluded	No statistical difference in terms of seasonal 25(OH)D levels of AS patients compared with HCs (30.79 ± 22.86 ng/ml versus 30.73 ± 18.53 ng/ml in the summer and 29.57 ± 30.47 ng/ml versus 29.82 ± 19.19 ng/ml in the winter)

Durmus et al., 2012, Turkey [19]	Insufficiency 20.1–29.9 ng/ml Deficiency ≤ 20 ng/ml	99	42	Yes	Yes	Yes	Excluded	No statistical difference in serum 25(OH)-D_3_ in AS patients compared with HCs (26.8 ± 11.7 ng/ml versus 31.1 ± 15.5 ng/ml, *p* = 0.073)

Mermerci Başkan et al., 2010, Portugal [24]	NR	100	58	Yes	Yes	Yes	Not excluded	The mean serum 25(OH)-D_3_ was significantly lower in AS patients as compared to HCs (21.70 ± 12.17 mmol/l versus 32.70 ± 8.7 mmol/l, *p* < 0.0001)

Lange et al., 2005, Germany [25]	NR	58	58	Yes	Yes	No	Not excluded	Significant reduction of vitamin D metabolites in both AS patients with and without osteoporosis compared with HCs (23 ± 6 pg/ml versus 30 ± 9 pg/ml versus 43 ± 10 pg/ml, *p* < 0.05 for 1.25 vitamin D_3_ and 10 ± 7 ng/ml versus 25 ± 6 ng/ml versus 29 ± 7 ng/ml, *p* < 0.05 for 25-vitamin D_3_

Obermayer-Pietsch et al., 2003, Germany [26]	NR	104	54	Yes	Yes	Yes	Not excluded	Significant lower vitamin D levels in AS patients compared with HCs (*p* = 0.002)

Lange et al., 2001, Germany [27]	Normal range: 6–42 ng/ml	70	45	Yes	Yes	No	Not excluded	The mean serum 1,25(OH)_2_-D_3_ was significantly lower in AS patients as compared with HCs (31 ± 13 pg/ml versus 42 ± 13 pg/ml, *p* < 0.05) There was no significant difference in the mean serum 25(OH)-D_3_ in AS patients (22 ± 1) as compared to HCs (24 ± 11), *p* > 0.05

Franck and Keck, 1993, Germany [28]	NR	38	52	No	No	No	Not excluded	Mean vitamin D metabolite concentrations were in the normal range in both AS patients and HCs (comparative values not given)

AS: ankylosing spondylitis; BMI: body mass index; HCs: healthy controls; NR: not reported.

^§^Definition according to the level of serum 25(OH)D.

**Table 2:** Vitamin D (vitamin D_2_ and vitamin D_3_) treatment for vitamin D deficiency as recommended by the Endocrine Society for the treatment of vitamin D deficiency.

Life stage group	Loading dose	Maintenance dose
0-1 year	2000 IU/day or 50000 IU/week for 6 weeks	400–1000 IU/day
1–18 years	2000 IU/day or 50000 IU/week for at least 6 weeks	600–1000 IU/day
Adults	6000 IU/day or 50000 IU/week for 8 weeks	1500–2000 IU/day

IU: international units.

## Abbreviations

25(OH)D_3_: 25-Hydroxycholecalciferol
1,25-(OH)_2_D_3_: 1,25-Dihydroxycholecalciferol
AS: Ankylosing spondylitis
AIDS: Acquired immunodeficiency syndrome
BASDAI: Bath Ankylosing Spondylitis Disease Activity Index
BASFI: Bath Ankylosing Spondylitis Functional Index
BMD: Bone mineral density
CRP: C-reactive protein
CVD: Cardiovascular diseases
DC: Dendritic cells
DMARDs: Disease-modifying antirheumatic drugs
ESR: Erythrocyte sedimentation rate
HLA: Human leukocyte antigen
IL: Interleukin
IOM: Institute of Medicine
MHC: Major histocompatibility complex
NSAIDs: Nonsteroidal anti-inflammatory drugs
Th: T helper
TNF-*α*: Tumor necrosis factor alpha
TNFi: Tumor necrosis factor inhibitors
VDR: Vitamin D receptor.

## Data Access

*Availability of Data and Material*. Data sharing is not applicable to this article as no datasets were generated or analyzed during the current study.
